# A cGAN-based network for depth estimation from bronchoscopic images

**DOI:** 10.1007/s11548-023-02978-z

**Published:** 2023-08-10

**Authors:** Lu Guo, Werner Nahm

**Affiliations:** Institute of Biomedical Engineering, Karlsruhe Institute of Technology, Kaiserstraße 12, 76131 Karlsruhe, Germany

**Keywords:** Depth estimation, Conditional GANs, Bronchoscopy, Image-guided surgery

## Abstract

**Purpose:**

Depth estimation is the basis of 3D reconstruction of airway structure from 2D bronchoscopic scenes, which can be further used to develop a vision-based bronchoscopic navigation system. This work aims to improve the performance of depth estimation directly from bronchoscopic images by training a depth estimation network on both synthetic and real datasets.

**Methods:**

We propose a cGAN-based network Bronchoscopic-Depth-GAN (BronchoDep-GAN) to estimate depth from bronchoscopic images by translating bronchoscopic images into depth maps. The network is trained in a supervised way learning from synthetic textured bronchoscopic image-depth pairs and virtual bronchoscopic image-depth pairs, and simultaneously, also in an unsupervised way learning from unpaired real bronchoscopic images and depth maps to adapt the model to real bronchoscopic scenes.

**Results:**

Our method is tested on both synthetic data and real data. However, the tests on real data are only qualitative, as no ground truth is available. The results show that our network obtains better accuracy in all cases in estimating depth from bronchoscopic images compared to the well-known cGANs pix2pix.

**Conclusions:**

Including virtual and real bronchoscopic images in the training phase of the depth estimation networks can improve depth estimation’s performance on both synthetic and real scenes. Further validation of this work is planned on 3D clinical phantoms. Based on the depth estimation results obtained in this work, the accuracy of locating bronchoscopes with corresponding pre-operative CTs will also be evaluated in comparison with the current clinical status.

## Introduction

As an alternative to the electromagnetic navigation system [1] which is the state-of-the-art technique used for assisting diagnostic and interventional bronchoscopy, vision-based bronchoscopic navigation system helps to track bronchoscope with the advantages of low-cost, less impact from tissue deformations, and no requirement for additional equipment setup [2]. The localization of the bronchoscope with respect to the preprocedural CTs is realized by applying 2D-3D registration approaches. Some examples can be found in [3–6]. Among the approaches, recovering the 3D geometrical structure of the scene based on depth estimation from bronchoscopic images has been proven to be more robust to illumination and texture variations and to preserve the morphological scene information [7]. As a significant step of this approach, our work aims to develop a method for directly estimating depth from bronchoscopic images.

Compared to classical methods (e.g., shape from shading), supervised deep networks show outstanding performance on depth estimation from single images. Instead of local pixel-wise loss functions on which many networks rely, the conditional generative adversarial networks (cGANs) can learn a loss function for depth estimation, which allows the recovery of features that would generally be lost in other networks, and is more context-aware since the discriminator forces the generator to generate estimated depth maps which have indistinguishable realistic pixel configurations compared with ground truth depth maps [8]. For training such networks in the bronchoscopic application, training data consisting of real bronchoscopic image-depth pairs are needed but difficult to obtain. Thus, we propose our cGAN-based BronchoDep-GAN partially trained on synthetic data, including realistic-looking textured bronchoscopic image-depth pairs and virtual image-depth pairs ([7] argues that embedding virtual images in training the model delivers significantly better depth estimation results.). To adapt our model to real bronchoscopic scenes, we also include unlabelled real bronchoscopic images as training data in an unsupervised fashion. To our knowledge, this is the first trial to involve virtual and real bronchoscopic images in the training phase for depth estimation in bronchoscopy in supervised and unsupervised fashions, respectively.

**Fig. 1:**
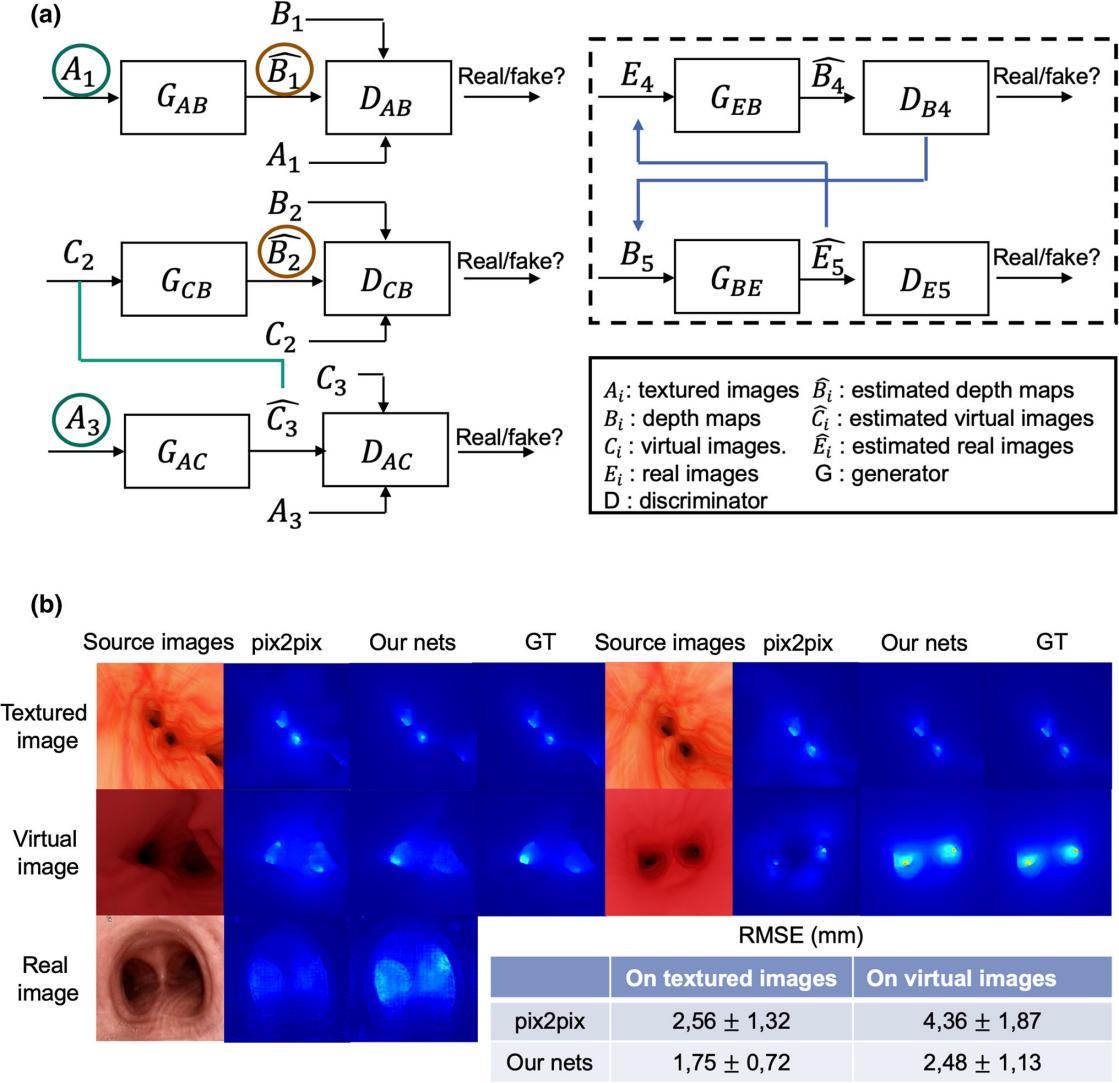
**a** Overview of BronchoDep-GAN; **b** comparison of depth estimation results using our nets and the well-known cGANs pix2pix

## Methods

### Data preparation

The synthetic bronchoscopic images in this work are generated using virtual bronchoscopy, which allows the creation of bronchoscope-like inner views of the human airway with data derived from CTs. They are divided into two groups, namely “textured images” and “virtual images” according to whether the images have realistic-looking colors and textures (which are generated by applying the spatial GAN proposed in [9, 10]). The corresponding depth maps of each synthetic image are rendered with a maximum depth of 15 cm.

For training the BronchoDep-GAN, approximately 1500 image-depth pairs from each synthetic data group and 1500 unpaired real bronchoscopic images and depth maps are used as training data.

### Depth estimation

The BronchoDep-GAN regards depth estimation as an image-to-image (bronchoscopic image-to-depth) translation task and is developed inspired by work [7]. Similarly, the virtual images are embedded into the training phase, but differently, in a supervised fashion. As is shown in the left side of Fig. 1a, this part includes three levels of paired image translation, i.e., textured image to depth map, virtual image to depth map, and textured image to virtual image translation. For each level, the architecture of the adversarial networks is adopted from pix2pix networks[11], which deal with paired image-to-image (source image to target image) translation task. The total loss from [11] which connects the GAN adversarial loss and pixel-wise L1 loss is applied to all three levels, and we refer to that of level i as $L_\textrm{pix2pixi}$. Here, the adversarial loss implies the learning strategy that the generator should be trained to fool the discriminator, whose task is to distinguish between the target images and the images generated by the generator from source images, whereas the L1 loss measures the pixel differences and tries to minimize the distortion of the generated images with reference to target images.

Besides, since the domain gap between synthetic and real images will lead to a performance drop when transferring the depth estimation models trained only on synthetic data to real scenes, real bronchoscopic images who have no corresponding depth maps are also embedded in the training phase of our network in an unsupervised fashion. This part translates between the domains of real bronchoscopic images and depth maps learning from unpaired data, and the network’s architecture (right side in Fig. 1a) is based on CycleGAN [12]. Here, apart from the GAN adversarial loss, the model encourages cycle consistency by adding an additional loss to measure the difference between the source images $E_4$ and the generated images $\hat{E_5}$ of the second generator$G_\textrm{BE}$ using $\hat{B_4}$ as input, and the reverse. This can be formally represented as $L_\textrm{cyc}(E,B) = \mathbb {E}_{E\sim p_\textrm{data}(E)}[\Vert E_4-G_\textrm{BE}(G_\textrm{EB}(E_4))\Vert _1]+\mathbb {E}_{B\sim p_\textrm{data}(B)}[\Vert B_5-G_\textrm{EB}(G_\textrm{BE}(B_5))\Vert _1]$, and further constrains the translations. Moreover, another loss function named identity loss, defined as $L_\textrm{identity}(E,B) = \mathbb {E}_{E\sim p_\textrm{data}(E)}[\Vert E_4-G_\textrm{BE}(E_4)\Vert _1]+\mathbb {E}_{B\sim p_\textrm{data}(B)}[\Vert B_5-G_\textrm{EB}(B_5)\Vert _1]$, is also included and helps to preserve color and tint in generated images. The total loss from [12] is applied to this part and is referred to as $L_\textrm{cycleGAN}$.

Furthermore, a merging loss function, which combines the supervised levels, is introduced and formulated as: $L_\textrm{m} = \left\| G_\textrm{AB}(A_1)-G_\textrm{CB}(G_\textrm{AC}(A_3))\right\| _{L2}$. It is designed to accumulate the benefits from supervised training of all three pairs. The total loss of our BronchoDep-GAN is then defined as: $L_\textrm{total} = L_\textrm{pix2pix} + \lambda _\textrm{cycleGAN}L_\textrm{cycleGAN} + \lambda _\textrm{m}L_\textrm{m}$, where $\lambda _\textrm{cycleGAN}$ and $\lambda _\textrm{m}$ represent weights of respective loss.

## Results

The trained model is tested on approximately 500 synthetic textured and virtual images, and the results are compared to that of the pix2pix model. Examples from the results and the quantitative evaluation are shown in Fig. 1b. Tests on real bronchoscopic images are also made. However, a quantitative evaluation is not possible in this case due to the lack of ground truth. We can tell that our nets produce better results in all cases (but only subjectively for real bronchoscopic images). In this case, our network predicts smoother depth maps that are more corresponding to the source images.

## Conclusion

Unlike previous approaches, virtual and real bronchoscopic images are embedded in the training phase of our proposed network, which enables better performance of depth estimation directly from both real and synthetic bronchoscopic images, compared to the well-known cGANs pix2pix. However, the lack of ground truth corresponding to real bronchoscopic images leads to a lack of quantitative evaluations of our method applied to real bronchoscopic images. In the future, tests will be introduced on 3D clinical airway phantoms, where images are acquired with a real bronchoscope, and the ground truth depths are rendered accordingly. The accuracy of tracking bronchoscope based on the depth estimation results from this work will also be evaluated in comparison with the current clinical status.

## Data Availability

Data used in this publication were generated by the National Cancer Institute Clinical Proteomic Tumor Analysis Consortium (CPTAC) [13, 14].
